# Effects of Cyberbullying Experience and Cyberbullying Tendency on School Violence in Early Adolescence

**DOI:** 10.2174/1874434601711010098

**Published:** 2017-08-10

**Authors:** Mi-Kyoung Cho, Miyoung Kim, Gisoo Shin

**Affiliations:** 1 Eulji University, Seongnam-si, South Korea; 2 Chung-Ang University, Seoul, South Korea

**Keywords:** Adolescent, Cyberbullying, Middle school, Violence’ ANOVA, smartphones, School violence

## Abstract

**Background:**

School violence in early adolescence, whose frequency and status have recently changed significantly.

**Objective:**

This study attempts to detect the cyber bullying inclination of youth in early adolescence when aggressiveness reaches its peak, to identify school violence, and to develop a school violence prevention program. Method: This study was a survey research, investigating participants who were 470 middle school students in South Korea. For the analysis, independent t-test, one-way ANOVA and hierarchical regression analysis.

**Results:**

It is suggested that the school violence victimization experience and cyber bullying infliction experience has an influence in the school violence infliction. And the cyber bullying victimization experience and school violence victimization experience variables exert effects.

**Conclusion:**

The results of this study suggest that school nurses who are connecting to the community-school-home should take an active part in the development of school violence mediation education program, considering the cultural characteristics of the country.

## 1 INTRODUCTION

Every morning, we wake up with the alarm from our smartphone, check the weather forecast for the day through the smartphone, and build human relationships by communicating with others through group chatting rooms. Smartphone is a small sized computer with mobile phone functions. After Apple released a smartphone which integrated three major functions such as iPod, mobile phone and mobile internet in 2007, we have experienced significant changes in our everyday life [1]. After Social Network Service (hereafter SNS) through smartphones has been activated, more than seven hundred million people have used Facebook or Twitter in the world and about half a billion text messages are being transferred per day through chatting rooms. SNS is an online platform which creates and enforces social relationships through free communication, information sharing and expansion of human relationships. The most important function of SNS is to create, maintain, enforce and expand social relationship network through this service [2]. The advantage of SNS is that you can build relationships with people all over the world through the internet unlike offline and share information without constraints of time and place. Despite the usefulness of SNS, however, the negative side of SNS is also spreading. One of the negative side of SNS is harassment.

Recently, cyber bullying can be defined as a behavior that causes physical and mental damage to others by infringing upon the honor or rights and interests of others through online space instead of offline. As cyber bullying is not restrained by time or space, multiple people can witness it at the same time, and it can expand at an unpredicted geometrical speed through the re tweet function of SNS such as 'Forward' and 'Recommend,' and it is called “Abuse in the 21^st^ Century.” [3]. Systematic studies on cyber bullying as a social phenomenon were initiated by Olweus in 1970s [4]. He defined cyber bullying as trials that cause harm or threat to a person with a similar meaning of aggressiveness which has been generally presented. Additionally, he said that cyber bullying is a new form of aggressiveness equivalent to crimes occurring in special environments and methods of school violence. Since it can maintain continuity and repeatability regardless of time and place by harassing victims remotely, it does not need physical proximity between the inflictor and the victim unlike in traditional school violence [4].

The age group which is badly affected by cyber bullying is the juvenile age group. In western countries, it has been reported that more than 25% of teenagers have experienced cyber bullying [5]. Youth are significantly affected, and it is reported that it can lead to maladjustment to school life and depression. It has also been reported that they can commit suicide more easily and frequently than adults [6]. It is generally known that youth are in the period of storm and stress, which causes physical, emotional and behavioral changes. Most importantly, the revelation of aggressiveness is presented as one of the psychological characteristics in adolescence [7]. Aggressiveness emerges in childhood, increases till middle of the adolescent period, and it reaches its peak in the early adolescent period. It has been shown that such aggressiveness is related to problematic behaviors such as drug use, misbehavior, and chronic school violence and cyber bullying experiences [8].

Another characteristic of cyber bullying among youth is that school violence is reciprocally and closely related to cyber bullying rather than it being totally separate from offline school violence [9]. School violence and cyber bullying occur due to inner motives such as ostentation of power, desire to govern or subdue others, revenge, boredom, jealousy and transition of emotions [10]. According to the general strain theory [11], school violence among youth occurs due to various tension factors that youth experience at home and at school in everyday life. In other words, youth develop negative emotions such as anger, depression and despair because of various tension factors and they execute school violent acts to vent these negative feelings [9]. However, not all youth who are exposed to tension and have negative feelings because of these tension factors commit school violence but school violence is related to ties with their family and school and their personal disposition. Factors affecting violence or misbehavior of youth have been reported as age, experience of harassment in the past, urge to control, impulsiveness, attitude toward aggressiveness and moral attitude such as pursuit of pleasure [12].

Accordingly, this study attempts to detect the cyber bullying inclination of youth in early adolescence to identify school violence, and to develop a school violence prevention program.

## 2 MATERIAL AND METHODS

### 2.1 Design

This study is a descriptive research to find out the cyber bullying experience, cyber bullying victimization, and cyber bullying tendency, and to examine the effects of cyber bullying experience and cyber bullying tendency on school violence.

### 2.2 Sample and Setting

Participants were 470 middle school students in S city in South Korea. For the selection of participants, G*power version 3.1.2 program was used. By calculating a two-tailed test significance level .05, medium effect size, power 0.95, and effect factors 17, and 305 participants were needed. We set the dropout rate as 35% from the calculated number of participants. From them, 440 copies of surveys were collected and used for the final analysis.

### 2.3 Variables and Instruments

#### 2.3.1 Cyber Bullying Infliction

In the questionnaire to assess cyber bullying experience, 4 questions about harassment through mobile phones were added to the existing questionnaire by Hinduja and Patchin [6] to develop a questionnaire based on 13 questions. Each question asked if they have been harassed or committed violence through the internet or mobile phone for the last one month and it was analyzed on a 5 point likert scale ranging from 1 ‘Never’ to 5 ‘Very much.’ The higher the score, the more likely the respondent was involved in the cyber bullying. The reliability of the original tool was Cronbach´α=.93, and in this study, it was Cronbach´α=.916.

#### 2.3.2 Cyber Bullying Victimization

To measure the cyber bullying victimization experience, total 11 questions were selected by adding 4 questions asking about harassment through mobile phones to the tool developed by Patchin and Hinduja [13]. Each question asked if they have been cyber harassed for the last one month and it was analyzed on a 5 point likert scale ranging from 1 point indicating ‘Never’ to 5 points indicating ‘Very much.’ The higher the score, the higher the respondent received the damage. The reliability of the original tool was Cronbach´α=.90, and in this study, it was Cronbach´α = .889.

#### 2.3.3 Cyber Bullying Tendency

The Cyber Bullying Tendency Diagnosis Tool by Yoon, Kim and Park [14] was developed and verified by defining cyber bullying according to the Korean situation by referring to overseas cyber bullying evaluation tools and previous related studies and analyzing the induction factors of cyber bullying. The sub-areas of the cyber bullying tendency diagnosis tool are cyber insulting, cyber stalking, cyber defamation, cyber sexual violence, cyber camouflage, cyber justification (4 questions in each area), cyber outcast (3 questions), and ethical attitude (2 questions). The total number of questions is 29. The tool uses a 4 step likert scale and the students were asked about the frequency of their experience in the last three months according to the scale. If they did not have any experience for the last three months, they would reply 1 'None.' They were asked to reply to 4 according to the frequency such as 'once or twice, many times a week, many times a month.' The higher the score, the higher was the cyberbullying tendency. The reliability of the original tool was Cronbach`α = .934, and in this study, it was Cronbach`α = .893.

#### 2.3.4 School Violence

As a school violence tool, the Olweus' [4] Offender/Victim Survey Scale was used. The tool is composed of 14 questions; 7 questions about school violence infliction experience and 7 questions about school violence victimization experience. Each question is analyzed on a 5point likert scale ranging from 1‘Not at all’ to 5‘Very Much Indeed.’ The higher was the number of points for the infliction experience and victimization experience, respectively, the higher was the level of school violence. The reliability of the original school violence infliction experience tool was Cronbach`α = .90, and in this study, it was Cronbach`α = .864. The reliability of the original school violence victimization experience tool was Cronbach`α = .91, and in this study, it was Cronbach`α = .835.

#### 2.3.5 Data Analysis

Collected materials were analyzed using the PASW Statistics 21.0 (Chicago, IL, USA) program. Characteristics of participants were analyzed using the average, standard deviation, frequency and percentage. The major variables of this study such as mental health, cyber bullying victimization and infliction experience, cyber bullying tendency, and school violence were analyzed using the average, standard deviation and range. The differences in major variables according to general characteristics were analyzed with independent t-test when there were two groups and one-way ANOVA was used when there were more than three groups. Post analysis was performed by Scheff**é** test. Correlations among mental health, cyber bullying infliction and victimization experience, cyber bullying tendency, and school violence were analyzed using Pearson's correlation coefficient. Effects of mental health, cyber bullying infliction and victimization experience, and cyber bullying tendency on school violence experience were analyzed using hierarchical regression analysis. The significance level of each statistic was considered at the level of *p*<0.05.

#### 2.3.6 Ethical Considerations

This study was approved by the Institutional Review Board of the university. Among all the students, 440 students understood the purpose and contents of this study and obtained their parents’ consent, and their data were collected. All participants participated voluntarily, and efforts were made to protect the participants’ human rights. The participants were informed that the data obtained through the questionnaire survey would not be used for any other purpose than for this study, that their personal information would be kept confidential, and that they could withdraw from the survey at any time.

## 3 RESULTS

### 3.1 Characteristics of Participants

Based on gender, there were 304 female students (69.1%), and their number was higher than that of male students. Based on the average time of internet use, 185 students (42.0%) used internet for two hours a day. The mean value of cyber bullying infliction and victimization was 1.18 (*S.D* 0.39) and 1.19 (*S.D* 0.47), respectively, and the number of students who experienced cyber bullying victimization was higher than the number of students who experienced cyber bullying infliction. The cyber bullying tendency in the participants was rated in the ascending order: psychological justification, cyber insulting, and cyber outcast (Table **1**).

### 3.2 Level of School Violence

The range of school violence infliction experience score was from 1 to 3, while the range of victimization experience was from 1 to 4.6, which shows that the victimization experience was more common than the infliction experience. The average value of school violence infliction was 1.11 (*S.D* 0.31), and the average value of victimization was 1.27 (*S.D* 0.53) (Table **2**).

### 3.3 School Violence According to the Participants Characteristics

With respect to the school violence infliction, grade of the participants (F=6.17, *p*=.002), cyber bullying infliction experience (t=-5.07, *p*<.001) and victimization experience (t=-4.24, *p*<.001) were found to be statistically significant. With respect to the school violence victimization, there was a statistically significant difference according to the family relationship that participants perceive (F=11.14, *p<*.001), satisfaction about school environments (F=30.61, *p<*.001), cyber bullying infliction experience (t=-5.46, *p*<.001), and victimization experience (t=-7.49, *p*<.001). With respect to the cyber bullying tendency, those who had points higher than the average had more number of experiences of both infliction and victimization than those who had below average points (Table **3**).

With respect to the cyber bullying infliction and victimization experience, and cyber bullying tendency, school violence experience showed a positive correlation with cyber bullying infliction experience (r=.43, *p*<.001), victimization experience (r=.26, *p*<.001) and cyber bullying tendency, out of which cyber bullying infliction experience showed the highest positive correlation. School violence victimization experience also showed a positive correlation with cyber bullying infliction experience (r=.35, *p*<.001), victimization experience (r=.43, *p*<.001), and cyber bullying tendency, and a high correlation was observed in the following order: school violence infliction experience and cyber bullying victimization experience (r=.46, *p*<.001) (Table **4**).

### 3.4 Effects of Cyber Bullying Infliction and Victimization Experience, and Cyber Bullying Tendency on School Violence

To identify the factors influencing school violence infliction and victimization experiences in the participants, a hierarchical multiple regression analysis was performed and its results are shown in Table (**5**). In model 1, categorical variables such as grade, sex, family relationship, and satisfaction about school environments were considered as dummy variables and average hours of internet use was entered as a continuous variable.

In model 2, a hierarchical multiple regression analysis was performed by including cyber bullying infliction experience, cyber bullying victimization experience, sub-areas of cyber bullying tendency, school violence infliction experience, and school violence victimization experience as independent variables, respectively. In the school violence infliction and victimization experience model, tolerance of independent variables in model 1 and 2 was more than 0.1, which was the standard, and both models satisfied the Variance Inflation Factor (VIF) to have lower than the standard (n=10). Therefore, they did not have the multi-collinearity problem.

In the school violence infliction experience model, factors influencing the school violence infliction experience in the participants were Grade 3 (*β* =-0.21, *p<*.001) and the average level of satisfaction about school environments (*β* =-0.20, *p*=.044) in model 1 and they explained 4% of the model (Adjusted *R*^2^=0.04, F=3.33, *p*=.001). In model 2, it was found that the cyber bullying infliction experience (*β* =0.14, *p*=.006), cyber defamation (*β* =0.14, *p*=.014), ethical attitude (*β* =0.13, *p*=.007) out of cyber bullying tendency, and school violence victimization variables (*β* =0.37, *p<*.001) had an influence. The total explanatory power was 31% and the school violence infliction experience model was statistically significant (Adjusted *R*^2^=0.31, F=11.08, *p*<.001).

In the school violence victimization experience model, factors influencing the school violence infliction experience in the participants were Grade 3 (*β* =-0.11, *p*=.048), family relationship (average: *β* =-0.19, *p*=.028, bad: *β* =-0.25, *p*=.005) and level of satisfaction about school environments (average: *β* =-0.65, *p<*.001, bad: *β* =-0.71, *p<*.001) in model 1 and they explained 14% of the model (Adjusted *R*^2^=0.14, F=9.63, *p*<.001). In model 2, it was found that the cyber bullying victimization experience (*β* =0.27, *p<*.001) and school violence infliction variables (*β* =0.33, *p<*.001) had an influence. The total explanatory power was 39% and the school violence victimization experience model was statistically significant (Adjusted *R*^2^=0.39, F=15.12, *p*<.001).

## 4 DISCUSSION

The results of this study showed that the cyber bullying experience and cyber bullying tendency affected school violence in early adolescence. Thus, the following findings are discussed:

Firstly, it was found that the school violence victimization experience and cyber bullying infliction experience has an influence in the school violence infliction model, which is the result of this study. According to a survey by the Statistics of South Korea 2014, school violence and cyber bullying are closely related. Especially, it was found that youths with the school violence victimization experience commit cyber bullying. Victims of school violence tend to have cyber bullying inflicting behaviors to reduce their tension for school violence. This is because cyber bullying has original characteristics unlike general school violence, such as anonymity, constancy, speed, diffusivity and visual shock. Especially, because of anonymity in cyber bullying, user tracing is difficult in mobile phones, email and SNS, and it is very difficult to identify bullying in smartphones compared to internet café, web sites or blogs. Therefore, victims of school violence can be transitioned into cyber bullying inflictors very easily. In a survey among 200 middle school students in USA, there was a positive relationship between victims who experienced school violence and cyber bullying inflictors 6 months later. This result shows the vicious circle in which victims in a peer group inflict violence upon others who are weaker than them and victims and inflictors overlap [15]. This shows that mediation is needed for both victims and inflictors.

However, in the school violence victimization experience model, it was found that the cyber bullying victimization experience and school violence victimization experience variables exert effects. The study results show that inflictors are few in number and majority are victims who are attacked, as shown in previous studies [9, 16]. In South Korea, the number of students who were arrested by police for being middle school violence inflictors in the last 3 years was 25,000 on average, which accounted for 1.5% of the total number of middle school students [17]. According to the Interactive Theory against school violence infliction behaviors [18], the school and social pathological phenomena are related. In other words, customary weakening of the social bond can be a cause of misbehavior. In case of inflictors, they participate in school violence to secure their social identity and status in the peer group and induce positive responses [19]. It is backed by various study results that present the pursuit of power as a cause of school violence, and it occurs more frequently in Asian countries such as China, Japan and South Korea rather than in western countries. It is based on the oriental culture which places greater importance on the group rather than on individuals [20].

On the other hand, the General Theory [20] out of the theories explaining school violence asserts that those who have strong self-control have a tendency to avoid violence and misbehaviors regardless of the situation and they can restrain from school violence by performing desirable behaviors controlling their own behaviors without external instructions or supervision. According to the General Theory [12], the self-control level in individuals is achieved at the age of 8 to 10 years and it is maintained for the rest of the life. Thus, people who have low self-control repeat inflicting violence. In the study by Turanovic and Pratt [21], the relationship between school violence infliction experience and self-control through the General Theory is presented. According to them, responses of individuals to school violence differ according to the level of self-control. In the school violence infliction experience model in this study, cyber bullying tendency of the youth had an influence on the school violence infliction experience. Among them, those who had a negative tendency against cyber defamation and ethical attitudes had school violence inflicting experiences. In a previous study, it was found that those who have a high level of moral and ethical deviation have more school violence infliction experiences [22]. To prevent school violence, it is necessary to improve the moral and ethical sensitivity of students from the elementary school days, to prevent deviation, and to develop teaching-learning activities that have an effect on the moral and ethical intensity.

For this purpose, various learning activities should be performed and the role of school nurses who are responsible for the physical and psychological health of students connecting the community, home, and school is very important. In this digital cyber era, cyber defamation tendency has become a social issue globally. Because of anonymity, non-face-to-face characteristics, reset syndrome, absence of community solidity, absence of time and space, and quick propagation, it has more serious results than offline physical school violence [15]. Youth can establish their identity when they become independent of their parents, they have their own activities, and they build relationships with others. In early adolescence, they need to vent out their frustration, and it is difficult and a chaotic era of storm and gale [23]. Therefore, a program should be developed for the cyberspace of the youth where they can express their dissatisfaction for and pressure from the school, home and friends. Especially, it is necessary to identify youth with a strong cyber defamation tendency at an early stage, and an in-school mediation program to mitigate their emotions should be initiated by school nurses.

The results of this study with respect to the connection between the school violence victimization experience and the school violence infliction experience are compatible with the results of previous studies. However, this study is meaningful as it showed the effects on cyber bullying tendency which previous studies could not demonstrate, but it also has a limitation as it assessed a part of the youth of a country (South Korea).

## CONCLUSION

This study was conducted to detect the cyber bullying inclination of youth in early adolescence to identify school violence in South Korea. On the results of this study, it is suggested that further studies are needed to assess the connection between cyber bullying tendency and school violence among youth in early adolescence. Also, school nurses who connect the community-school-home should take an active part in the development of the school violence mediation education program considering the cultural characteristics of the country.

## Figures and Tables

**Table 1:** Characteristics of the subjects (n=440).

Characteristics			N (%)	Mean±SD
Grade		1^st^ year student	132 (30.0)	
		2^nd^ year student	130 (29.5)	
		3^rd^ year student	178 (40.5)	
Gender		Male	136 (30.9)	
		Female	304 (69.1)	
Family relationship*		Good	319 (72.5)	
		Average	93 (21.1)	
		Bad	25 (5.7)	
Satisfaction of school life*		Good	266 (60.5)	
		Average	144 (32.7)	
		Bad	27 (6.1)	
Average internet use time (hours)*		No use	91 (20.7)	1.81±2.25
		< 2	154 (35.0)	
		≥ 2	185 (42.0)	
Cyberbullying infliction experience		<1.18	343 (78.0)	1,18±0.39
		≥1.18	97 (22.0)	
Cyberbullying victimization experience		<1.19	313 (71.1)	1.19±0.47
		≥1.19	127 (28.9)	
Cyberbullying	Insulting	<1.18	293 (66.6)	1.18±0.36
tendency		≥1.18	147 (33.4)	
	Stalking	<1.09	354 (80.5)	1.09±0.25
		≥1.09	86 (19.5)	
	Defamation	<1.10	339 (77.0)	1.10±0.25
		≥1.10	101 (23.0)	
	Sexual violence	<1.08	362 (82.3)	1.08±0.20
		≥1.08	78 (17.7)	
	Camouflage	<1.15	308 (70.0)	1.15±0.30
		≥1.15	132 (30.0)	
	Outcast	<1.16	316 (71.8)	1.16±0.34
		≥1.16	124 (28.2)	
	Justification*	<1.26	319 (72.5)	1.26±0.44
		≥1.26	118 (26.8)	
	Ethical attitude*	<1.10	385 (87.5)	1.10±0.30
		≥1.10	54 (12.3)	

*Missing, SD: Standard Deviation

**Table 2:** Means of school violence (n=440).

Variables		Mean	Standard Deviation	Min-Max
School violence	Infliction experience	7.79	2.14	7-21
	Victimization experience	8.92	3.72	7-32

**Table 3:** Differences in school violence by characteristics of participants (n=440).

Characteristics			School violence					
			Infliction Experience			Victimization Experience		
			M±SD	t or F	*p*	M±SD	t or F	*p*
Grade		1^st^	8.24±2.51^a^	6.17	.002* (a>b)	9.27±4.07	0.91	.402
		2^nd^	7.88±2.32^ab^			8.84±3.61		
		3^rd^	7.40±1.57^b^			8.71±3.53		
Gender		Male	8.06±2.56	1.57	.119	8.76±3.36	-0.60	.550
		Female	7.67±1.92			8.99±3.87		
Family relationship		Good	7.67±1.93	2.30	.102	8.52±3.14^a^	11.14	<.001* (a<b)
		Average	8.04±2.61			9.52±4.44^a^		
		Bad	8.44±2.76			11.84±5.85^b^		
Satisfaction of school life		Good	7.70±1.90	2.73	.066	8.40±2.83^a^	30.61	<.001* (a<b)
		Average	7.81±2.14			8.88±3.18^a^		
		Bad	8.70±3.80			13.85±7.84^b^		
Average internet use time (hours)		No use	7.78±2.44	1.43	.240	9.56±4.39	2.94	.054
		< 2	7.60±1.84			8.42±2.93		
		≥ 2	7.99±2.26			9.10±3.98		
Cyberbullying infliction experience		<15.30	7.39±1.32	-5.07	<.001	8.24±2.81	-5.46	<.001
		≥15.30	9.22±3.48			11.29±5.29		
Cyberbullying victimization experience		<13.13	7.44±1.42	-4.24	<.001	7.89±2.29	-7.49	<.001
		≥13.13	8.67±3.15			11.43±5.12		
Cyberbullying	Insulting	<4.73	7.47±1.80	-4.01	<.001	8.33±4.28	-4.37	<.001
tendency		≥4.73	8.43±2.59			10.08±3.62		
	Stalking	<4.38	7.48±1.68	-4.57	<.001	8.49±3.38	-4.23	<.001
		≥4.38	9.08±3.14			10.67±4.50		
	Defamation	<4.39	7.41±1.63	-5.33	<.001	8.47±3.34	-4.02	<.001
		≥4.39	9.07±2.99			10.41±4.84		
	Sexual violence	<4.31	7.66±2.08	-2.58	.011	8.67±3.56	-2.96	.003
		≥4.31	8.40±2.33			10.04±4.23		
	Camouflage	<4.62	7.53±1.83	-3.50	.001	8.35±3.18	-4.34	<.001
		≥4.62	8.41±2.63			10.23±4.50		
	Outcast	<3.48	7.44±1.55	-4.37	<.001	8.36±3.18	-4.39	<.001
		≥3.48	8.69±3.01			10.32±4.56		
	Justification	<5.04	7.41±1.51	-4.86	<.001	8.29±2.97	-4.96	<.001
		≥5.04	8.85±3.08			10.66±4.87		
	Ethical attitude	<2.20	7.54±1.69	-4.10	<.001	8.61±3.34	-3.41	.001
		≥2.20	9.61±3.66			11.13±5.29		

* Post-Hoc: Scheffé test

**Table 4:** Correlations of cyberbullying infliction and victimization experience, cyberbullying tendency, and school violence (n=440).

Variables		CI	CV	CT								SV^2^	
				I	S	D	SV^1^	C	O	J	E	IE	VE
		r (*p*)											
CI		1											
CV		.66 (<.001)	1										
CT	I	.45 (<.001)	.34 (<.001)	1									
	S	.40 (<.001)	.27 (<.001)	.63 (<.001)	1								
	D	.40 (<.001)	.30 (<.001)	.59 (<.001)	.68 (<.001)	1							
	SV^1^	.19 (<.001)	.18 (<.001)	.31 (<.001)	.37 (<.001)	.41 (<.001)	1						
	C	.35 (<.001)	.27 (<.001)	.50 (<.001)	.54 (<.001)	.54 (<.001)	.52 (<.001)	1					
	O	.39 (<.001)	.30 (<.001)	.48 (<.001)	.44 (<.001)	.56 (<.001)	.30 (<.001)	.62 (<.001)	1				
	J	.34 (<.001)	.24 (<.001)	.47 (<.001)	.38 (<.001)	.49 (<.001)	.24 (<.001)	.53 (<.001)	.60 (<.001)	1			
	E	.23 (<.001)	.17 (<.001)	.26 (<.001)	.32 (<.001)	.27 (<.001)	.19 (<.001)	.32 (<.001)	.32 (<.001)	.55 (<.001)	1		
SV^2^	IE	.43 (<.001)	.30 (<.001)	.32 (<.001)	.35 (<.001)	.44 (<.001)	.16 (<.001)	.28 (<.001)	.45 (<.001)	.40 (<.001)	.37 (<.001)	1	
	VE	.35 (<.001)	.55 (<.001)	.24 (<.001)	.26 (<.001)	.23 (<.001)	.13 (<.001)	.22 (<.001)	.27 (<.001)	.32 (<.001)	.23 (<.001)	.46 (<.001)	1

CI: Cyberbullying infliction experience, CV: Cyberbullying victimization experience, CT: Cyberbullying tendency,

SV^2^: School violence, IE: Infliction experience, VE: Victimization experience, I: Insulting, S:Stalking, D:Defamation,

SV^1^: Sexual violence, C: Camouflage, O: Outcast, J: Justification, E: Ethical attitude

**Table 5:** Influencing factors on school violence (n=400).

Variables		School Violence Infliction Experience						School Violence Victimization Experience					
		Model 1			Model 2			Model 1			Model 2		
		β	t	*p*	β	t	*p*	β	t	*p*	β	t	*p*
Constant		-	15.51	<.001	-	3.81	<.001	-	15.44	<.001	-	4.41	<.001
Grade (ref=1st year)													
2nd year		-0.11	-1.83	.068	-0.05	-1.00	.322	-0.05	-0.96	.337	0.004	-0.08	.934
3rd year		-0.21	-3.71	<.001	-0.12	-2.38	.018	-0.11	-1.98	.048	-0.02	-0.49	.626
Gender (ref=Male)													
Female		-0.09	-1.76	.079	-0.08	-1.71	.088	0.04	0.75	.454	0.04	1.05	.296
Family relationships (ref=Good)													
Average		-0.08	-0.91	.362	0.001	0.01	.988	-0.19	-2.21	.028	-0.21	-2.81	.005
Bad		-0.15	-1.55	.122	-0.02	-0.23	.821	-0.25	-2.85	.005	-0.22	-2.84	.005
Satisfaction of school life (ref=Good)													
Average		-0.20	-2.02	.044	0.12	1.27	.204	-0.65	-6.84	<.001	-0.47	-5.68	<.001
Bad		-0.20	-1.90	.058	0.14	1.50	.135	-0.71	-7.21	<.001	-0.50	-5.85	<.001
Average internet use time (hours)		0.05	1.02	.310	0.02	0.44	.663	-0.01	-0.16	.877	-0.04	-1.11	.268
CI					0.14	2.78	.006				0.01	0.20	.845
CV					-0.02	-0.46	.643				0.27	5.89	<.001
CT	I				-0.07	-1.29	.197				-0.01	-0.17	.869
	S				0.07	-1.35	.177				0.03	0.65	.514
	D				0.14	2.47	.014				-0.08	-1.45	.148
	SV^1^				0.02	0.36	.716				-0.03	-0.59	.553
	C				-0.08	-1.40	.162				0.06	1.13	.257
	O				0.05	0.95	.340				0.04	0.87	.383
	J				0.05	0.83	.406				0.09	1.83	.068
	E				0.13	2.71	.007				-0.01	-0.23	.822
SV^2^	IE										0.33	7.55	<.001
	VE				0.37	7.55	<.001						
F (*p)*		3.33 (.001)			11.08 (<.001)			9.63 (<.001)			15.12 (<.001)		
Adj R^2^		0.04			0.31			0.14			0.39		
Tolerance		0.21-0.97			0.18-0.94			0.21-0.97			0.20-0.94		
VIF		1.03-4.80			1.07-5.49			1.03-4.80			1.06-5.09		
Durbin-Watson					2.02						1.90		

CI: Cyberbullying infliction experience, CV: Cyberbullying victimization experience, CT: Cyberbullying tendency,

SV^2^: School violence, IE: Infliction experience, VE: Victimization experience, I: Insulting, S:Stalking, D:Defamation,

V^1^: Sexual violence, C: Camouflage, O: Outcast, J: Justification, E: Ethical attitude
